# Deciphering Serous Effusions Using the New International System for Reporting Serous Fluid Cytopathology

**DOI:** 10.7759/cureus.60042

**Published:** 2024-05-10

**Authors:** Harika Mandava, Inuganti Venkata Renuka, Ramya Potti, Bellamkonda Mounica, Indurekha Kalla

**Affiliations:** 1 Pathology, NRI Medical College, Guntur, IND

**Keywords:** international system for reporting serous fluid cytopathology, cell block, diagnostic test accuracy, conventional smear, risk of malignancy, serous effusions

## Abstract

Introduction

Serous effusion cytopathology is a minimally invasive, cost-effective procedure and plays a crucial role in diagnosing a spectrum of pathological conditions, ranging from benign to malignant. The International System for Reporting Serous Fluid Cytopathology (ISRSFC) offers a standardized framework for reporting serous effusions, aiding in better communication and clinical decision-making.

Aims and objectives

This study aimed to categorize effusions using the ISRSFC reporting system. In addition, we sought to estimate the risk of malignancy (ROM) for each diagnostic category and evaluate the diagnostic performance of conventional smear versus cell block techniques.

Materials and methods

This cross-sectional study was conducted in the Department of Pathology over one year. We applied the ISRSFC criteria to serous effusions and categorized them accordingly. The ROM for each category was assessed with histopathology serving as the gold standard. Then, the diagnostic performance including sensitivity, specificity, positive predictive value (PPV), negative predictive value (NPV), and diagnostic accuracy was evaluated using conventional smear and cell block techniques.

Results

The study included 185 serous effusion cases, with ages ranging from two months to 85 years. The male-to-female ratio was 1.1:1. Most effusions were pleural fluids constituting about 133 cases (71.9%), followed by peritoneal fluids (47 cases, 25.4%) and pericardial fluids (five cases, 2.7%). Among the fluids, four (2.2%) were diagnosed as non-diagnostic (ND), 152 (82.2%) as negative for malignancy (NFM), four (2.2%) as atypia of undetermined significance (AUS), nine (4.8%) as suspicious for malignancy (SFM), and 16 (8.6%) as malignant (MAL). The overall ROM was 25% for ND, 8.5% for NFM, 50% for AUS, 77% for SFM, and 100% for MAL. The sensitivity, negative predictive value (NPV), and diagnostic accuracy were superior when combining conventional smear with the cell block technique.

Conclusions

Our findings underscore the use of ISRSFC in categorizing effusion samples, assessing the ROM, and guiding clinical management. Moreover, our study highlights the benefits of employing a combined approach using conventional smears and cell blocks for enhanced diagnostic accuracy in serous effusions.

## Introduction

Serous effusions found in the pleural, peritoneal, and pericardial cavities constitute a significant portion of cytology samples encountered in routine practice [1]. It is a simple, minimally invasive, and cost-effective procedure that aids in patient management [2]. These fluids represent myriad pathological conditions, ranging from non-neoplastic to neoplastic origin, each exhibiting its unique cellular composition [3]. However, interpreting serous fluid cytology poses challenges, including reactive cellular changes in mesothelial cells, and morphological similarities between malignant and mesothelial cells [4-6]. Thus, accurate reporting of cytopathologic findings remains critical for guiding effective clinical management strategies for patients.

In 2020, the International System for Reporting Serous Fluid Cytopathology (ISRSFC) emerged as the pioneering effort to establish a tiered structure for standardized reporting terminology of serous effusions. Mirroring other diagnostic cytology reporting systems, the ISRSFC comprises five diagnostic categories. They are categorized as non-diagnostic (ND), negative for malignancy (NFM), atypia of undetermined significance (AUS), suspicious for malignancy (SFM), and malignant (MAL). This system not only suggests when to employ specific terminologies but also outlines diagnostic criteria and aids in clinical management strategies [1,7-9].

In the present study, we collected serous effusion fluids from the pleural, peritoneal, and pericardial over one year in the cytopathology laboratory and applied the ISRSFC reporting system. Our study stands out as one of the few in the literature aimed at categorizing effusions, assessing the risk of malignancy (ROM), and evaluating the diagnostic performance of various preparatory methods, including conventional smears and cell block, in terms of sensitivity, specificity, positive predictive value (PPV), negative predictive value (NPV), and diagnostic accuracy.

## Materials and methods

Data retrieval

This cross-sectional study was conducted in the cytology section of the Department of Pathology at NRI Medical College and General Hospital, Chinakakani, India, from January 2022 to December 2022, following approval from the Institutional Ethics Committee. The study encompassed all serous effusion samples received in the cytology laboratory. Comprehensive demographic, clinical, radiological, and histopathological details were documented from the medical records. Fluids with inadequate volume (<50 ml), peritoneal washings, and those lacking complete clinical and radiological information were excluded from the study.

Processing the sample

The effusion sample was divided into two equal portions and subjected to centrifugation at 2500 rpm for 15 minutes, and the supernatant was discarded. One portion was utilized for conventional smear preparation, while the other was allocated for cell block preparation. For conventional smear preparation, two smears were made from the sediment and promptly fixed in ethanol. Following fixation, they were stained with Papanicolaou stain and hematoxylin and eosin (H&E). For cell block preparation, the supernatant fluid was carefully removed, leaving behind the residual cell pellet. This pellet was then fixed in freshly prepared Bouin's solution, consisting of saturated picric acid, glacial acetic acid, and formalin. After fixation, the cell pellet was processed and embedded in a paraffin block. Sections of 4-5 μ thickness were cut and stained with routine H&E. The slides were then dried, mounted, and studied under the microscope.

Diagnostic categorization

All the cases were classified according to the ISRSFC criteria [7]. In cases of discrepancy especially those in the indeterminate categories, two cytopathologists discussed, and a consensus was reached on the final diagnosis. They were classified as 1) non-diagnostic (ND) (effusions with insufficient cellularity or excess degeneration and obscured by blood), (2) negative for malignancy (NFM) (effusions that completely lacked evidence of malignancy; the cellular morphology included mesothelial cells, lymphocytes, macrophages, and polymorphs); (3) atypia of undetermined significance (AUS) (effusions with minimal architectural and cellular atypia comprising a spectrum from reactive atypia to degenerated tumor cells); 4) suspicious for malignancy (SFM) (effusions with features of malignancy but insufficient either in quality or quantity for a clearcut diagnosis of malignancy); and 5) malignancy (MAL) (effusions with cytomorphologic features of malignancy).

Statistical analysis

The data were entered into an Excel spreadsheet and analyzed. The percentages, ratios, and median values were calculated. The ROM for each category of ISRSFC was assessed. The diagnostic performance parameters like sensitivity, specificity, PPV, NPV, and diagnostic accuracy of conventional smear and cell block were calculated. The ROM assessment and evaluation of diagnostic performance analysis were calculated using histopathology as the gold standard. For cases without histopathology, clinical and radiological follow-up was done for one year to exclude malignancy. The ROM was calculated for each category as the ratio of the number of malignant cases confirmed histologically to the total number of cases in the diagnostic category. For accurate diagnostic parameter evaluation, MAL and SFM cases were considered positive, while NFM and AUS were considered negative for malignancy. Effusions lacking cell block and ND cytology samples were excluded from the performance analysis as they could not be classified as either positive or negative for malignancy. Considering histopathology as the gold standard, the sensitivity, specificity, PPV, NPV, and diagnostic accuracy were calculated using the following formulas:

Sensitivity = True positive/(True positive+False negative) x 100. Specificity = True negative/(True negative+False positive) x 100. PPV = True positive/(True positive+False positive) x 100. NPV = True negative/(True negative+False negative) x 100. Diagnostic accuracy = (True positive+True negative)/Total number of cases x 100.

## Results

**Figure 1:**
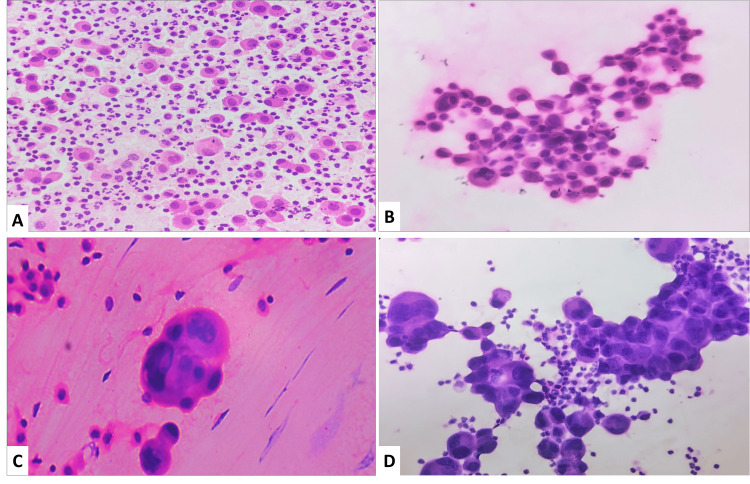
Cytological images of individual category A. Negative for Malignancy (NFM): comprising mesothelial cells, macrophages, lymphocytes, and neutrophils (H&E 400x). B. Atypia of undetermined significance (AUS) with cells showing mild anisonucleosis (H&E 400x). C. Suspicious for malignancy (SFM) atypical cell cluster with high N:C ratio and irregular nuclear membrane and abundant cytoplasm (H&E 400x). D. Malignancy (MAL) pleomorphic cells in glandular patterns and clusters with a high N:C ratio with few inflammatory cells (H&E 400x).

The study comprised 185 serous effusions, spanning ages from two months to 85 years with a median age of 49 years. The male-to-female ratio was 1.1:1, indicating a slight male predominance. Pleural effusions were the predominant type among the effusion samples, comprising 133 (71.9%) cases, followed by peritoneal effusions and pericardial effusions accounting for 47 (25.4%) and five (2.7%), respectively. Of all specimens, four (2.2%) were diagnosed as ND, 152 (82.2%) as NFM, four (2.2%) as AUS, nine (4.8%) as SFM, and 16 (8.6%) as MAL, listed in Table 1. The conventional smear images for each category are shown in Figure 1.

**Table 1:** Distribution of effusion cases based on age, sex, and diagnostic category No.: number, ND: non-diagnostic, NFM: negative for malignancy, AUS: atypia of undetermined significance, SFM: suspicious for malignancy, MAL: malignancy

Parameter	Pleural effusion	Peritoneal effusion	Pericardial effusion	Total
Age	2 months-85 years	22-73 years	21-50 years	2 months-85 years
Males	73	23	2	98
Female	60	24	3	87
Male: female ratio	1.2:1	1:1.04	1:1.5	1.1:1
No. of cases with cell block	125	26	3	154
ND category cases	3	1	0	4
NFM category cases	115	34	3	152
AUS category cases	2	2	0	4
SFD category cases	5	2	2	9
MAL category cases	8	8	0	16
Total no. of cases	133	47	5	185

**Figure 2:**
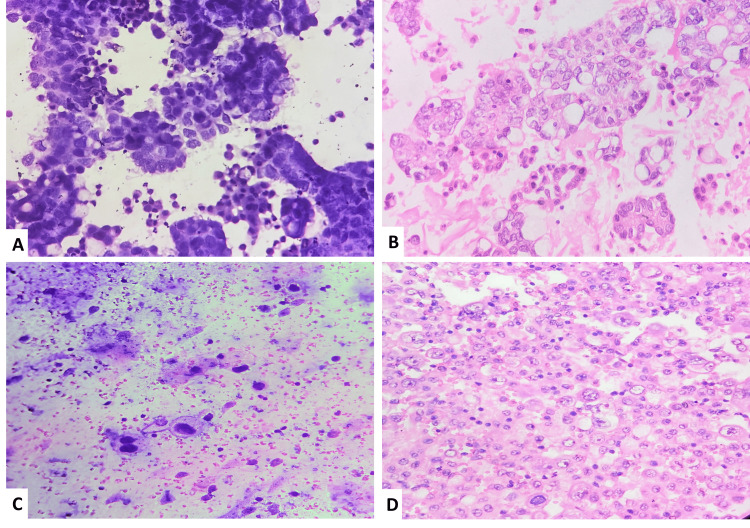
Conventional smear and cell block correlation of malignant effusions A. Conventional smear of malignant peritoneal effusion from ovarian carcinoma showing glandular and papillary patterns of malignant epithelial cells (H&E 400x). B. Cell block of the same case showing glandular architecture of tumor cells with pleomorphic vesicular nuclei and a moderate amount of eosinophilic to vacuolated cytoplasm (H&E 400x). C. Conventional smear of malignant pleural effusion from carcinoma lung showing atypical squamous cells with high N:C ratio and abundant cytoplasm (Papanicolaou stain 200x). D. Cell block of the same showing sheets of malignant squamous epithelial cells with hyperchromatic to vesicular nuclei and few showing prominent nucleoli and abundant eosinophilic cytoplasm (H&E 400x).

The overall ROM varied across categories, 25% (1/4) for ND, 8.5% (13/152) for NFM, 50% (2/2) for AUS, 77% (7/9) for SFM, and (16/16) 100% for MAL. The ROM among various serous effusions is summarized in Table 2. Cell blocks (CBs) were available in 154 cases (83.2%). Figure 2 shows the conventional smear and CB correlation of malignant effusions.

**Table 2:** Risk of malignancy assessment among various serous effusions ND: non-diagnostic, NFM: negative for malignancy, AUS: atypia of undetermined significance, SFM: suspicious for malignancy, MAL: malignancy, ROM: risk of malignancy, No.: number

Category	ND	NFM	AUS	SFM	MAL
No. of malignant cases	2	13	2	7	16
Pleural effusions %ROM	33.3%	9.5%	50%	60%	100%
Peritoneal effusions %ROM	0%	5.8%	50%	100%	100%
Pericardial effusions %ROM	-	0%	-	100%	-
Overall %ROM	25%	8.5%	50%	77%	100%

In this study, we analyzed 133 cases of pleural effusions, spanning ages from two months to 85 years, with a median age of 53 years. Males comprised 73 cases, while females constituted 60 cases, resulting in a male-to-female ratio of 1.2:1. Among these cases, three (2.2%) were classified as ND, 115 (86.5%) as NFM, two (1.5%) as AUS, five (3.8%) as SFM, and eight (6.0%) as MAL. CBs were available in 125 cases (93.9%). The ROM varied across categories, with ND at 33.3%, NFM at 9.5%, AUS at 50%, SFM at 60%, and MAL at 100%.

Our analysis included 47 cases of peritoneal effusions, with ages ranging from 22 to 73 years with a median age of 47 years. There were 23 male and 24 female patients, resulting in a male-to-female ratio of 1:1.04. Among these cases, one case (2.1%) was ND, 34 (72.3%) were NFM, 2 (4.2%) were AUS, 2 (4.2%) were SFM, and 8 (17.0%) were MAL. CBs were available in 26 cases (55.3%). ROM varied across categories, with ND at 0%, NFM at 5.8%, AUS at 50%, SFM at 100%, and MAL at 100%.

We observed five cases of pericardial effusion in our study, with ages ranging from 21 to 50 years and a median age of 36 years. Among these cases, there were two males and three females, resulting in a male-to-female ratio of 1:1.5. Among the five cases, three (60%) were diagnosed as NFM and two (40%) as SFM. CBs were available in three cases (60%). Given the absence of ND, AUS, and MAL cases, ROM was calculated only for NFM and SFM, resulting in 0% for NFM and 100% for SFM.

The diagnostic performance metrics for detecting malignancy in serous effusion cases varied across different diagnostic methods, as listed in Table 3. Conventional smears (CS) demonstrated a sensitivity of 60.53% and a specificity of 98.60%, with a PPV of 92% and an NPV of 90.38%, resulting in an overall diagnostic accuracy of 90.61%. On the other hand, the CB showed slightly higher sensitivity at 71.05% but lower specificity at 97.35%, with similar PPV and NPV values of 90% and 90.91%, respectively, leading to a diagnostic accuracy of 90.73%. Combining both CS and CB improved the sensitivity to 71.05% while maintaining a specificity of 97.90%, resulting in a higher PPV of 90.0% and an improved NPV of 92.72%, with an overall diagnostic accuracy of 92.27%. These findings underscore the importance of utilizing complementary diagnostic methods to enhance the accuracy of malignancy detection in serous effusion cytology.

**Table 3:** Diagnostic performance of the conventional smear and cell block PPV: positive predictive value, NPV: negative predictive value

Parameter	Conventional smear	Cell block	Combined smear and cell block
Sensitivity	60.53%	71.05%	71.05%
Specificity	98.60%	97.35%	97.90%
PPV	92.0%	90.0%	90.0%
NPV	90.38%	90.91%	92.72%
Diagnostic accuracy	90.61%	90.73%	92.27%

## Discussion

Serous effusion cytology serves as a minimally invasive and cost-effective diagnostic tool to explore the causes of body cavity effusions, offering valuable insights for clinical decision-making. We enrolled 185 fluid samples in this study and applied the recently proposed ISRSFC criteria. The age spanned from two months to 85 years. This aligns with the findings of Kundu et al. [10], whose study observed ages ranging from seven months to 92 years. Our study reported a male-to-female ratio of 1.1:1, similar to the ratio reported by Sun et al., with a slight male preponderance [11]. The predominance of pleural effusions, followed by peritoneal and pericardial effusions, aligns with the findings reported by Pergaris et al. [6].

Among the cases examined, 2.2% were diagnosed as ND, due to scant cellularity, excess degenerative cells, or obscuring blood. The majority, 82.2%, constituted NFM cases, suggesting a reactive process with mesothelial proliferations, lymphocyte-rich effusions, and mixed inflammatory cells. AUS comprised 2.2%, indicating ambiguous cell characteristics. In addition, 4.8% were categorized as SFM, suggesting potential malignancy, while 8.6% were definitively diagnosed as MAL, indicating the presence of malignant cells. Our findings were similar to the other cohorts in the literature [2,6,10,12-14]. In contrast to our study, a study by Zhu et al. reported a higher malignancy rate of 47.8%. This could be attributed to their study setting in an oncology center where cases were predominantly neoplastic [15]. Our findings, which were from a general hospital, reflect a higher proportion of NFM diagnoses pointing to a non-neoplastic reactive etiology and correlated with other studies of similar settings [6,11,12].

In our study, the overall ROM was 25% for ND, 8.5% for NFM, 50% for AUS, 77% for SFM, and 100% for MAL. These results were consistent with the ROM figures across different studies by Kundu et al. and Xu et al. Their reported ROM for different diagnostic categories were 20% and 26.7% for ND, 12% and 16.7% for NFM, 50% and 62.3% for AUS, 77.8% and 94.4% for SFM, and 100% for MAL [10,16]. This alignment underscores the reliability and reproducibility of diagnostic outcomes across multiple researchers, enhancing confidence in the reported findings.

ROM assessments for pleural and peritoneal fluids were compared with findings from other studies, as detailed in Table 4. The ROM values for diagnostic categories aligned closely with those reported in prior research [11,12,17]. However certain discrepancies, notably in the SFM category for pleural effusions, with ROM rates lower in our study compared to others were observed. This difference could be attributed to the potential instances of over-diagnosing reactive atypia cases as SFM in our study. For pericardial effusions, the ROM for NFM was 0%, consistent with the findings of Ahuja et al. [12]. For SFM, it is 100%, mirroring the results of the study conducted by Zhu et al. [15]. Although inconsistencies in reports among various studies were inevitable due to variations in the cases and clinical practices among different institutions, we believe that ROM assessment provides valuable and significant information to clinicians within an individual institution.

**Table 4:** Comparison of ROM in pleural and peritoneal effusions among various studies ND: non-diagnostic, NFM: negative for malignancy, AUS: atypia of undetermined significance, SFM: suspicious for malignancy, MAL: malignancy, n*:* number of cases

Category	Current study		Straccia et al., 2022 [17]		Ahuja and Malviya, 2022 [12]		Sun et al., 2022 [11]	
	Pleural n = 133	Peritoneal n = 47	Pleural n = 1292	Peritoneal n = 2257	Pleural n = 831	Peritoneal * *n = 457	Pleural n = 359	Peritoneal* *n = 217
ND	33.3%	0%	18.5%	19.3%	0%	50.0%	11.1%	18.2%
NFM	9.5%	5.8%	12.0%	15.0%	2.1%	4.8%	3.6%	1.8%
AUS	50%	50%	45.3%	43.5%	33.3%	22.2%	55.6%	55.5%
SFM	60%	100%	93%	100%	94.1%	83.3%	83.3%	100%
MAL	100%	100%	100%	100%	100%	100%	100%	100%

The diagnostic performance parameters for conventional smears in detecting malignancy, including sensitivity, specificity, PPV, NPV, and diagnostic accuracy, were 60.53%, 98.60%, 92%, 90.38%, and 90.61%, respectively. Corresponding values for cell block were 71.05%, 97.35%, 90%, 90.91%, and 90.73%. Combining conventional smear and cell block yields sensitivity, specificity, PPV, NPV, and diagnostic accuracy of 71.05%, 97.90%, 90.0%, 92.72%, and 92.27%, respectively. The combined approach demonstrated superior sensitivity, NPV, and diagnostic accuracy compared to using conventional smear or cell block alone. These findings were consistent with interpretations by Zhu et al. and Matreja et al. [15,18]. Hence, employing a combined approach using two preparatory methods enhances better diagnostic categorization and optimizes patient care. Additionally, cell block preparations can be used for other ancillary testing like immunohistochemistry (IHC).

The study's limitations stem from its small sample size and the absence of simultaneous biopsies of the pleura, peritoneum, and pericardium for cytohistological correlation. Consequently, due to this constraint, surgical biopsies of the primary tumor were predominantly used for correlation. This approach is justifiable because analyzing effusion cases followed by biopsies might artificially inflate the ROM, given that such biopsies are typically reserved for cases with a strong clinical suspicion of malignancy. Furthermore, as the study was conducted in a general hospital setting, there may be inherent differences compared to reports from specialized oncological hospitals, potentially introducing discrepancies.

## Conclusions

Serous effusion cytology is a simple, minimally invasive procedure. The newly proposed ISRSFC provides high accuracy and strategizes patient management. It provides easy communication with clinicians in terms of adequacy and ROM assessment for each category and helps in better patient care. Our study proposes the combined use of conventional smear and cell block to provide better sensitivity, NPV, and diagnostic accuracy than using one preparatory method alone.
